# Phospho-Ser^784^-VCP Drives Resistance of Pancreatic Ductal Adenocarcinoma to Genotoxic Chemotherapies and Predicts the Chemo-Sensitizing Effect of VCP Inhibitor

**DOI:** 10.3390/cancers13205076

**Published:** 2021-10-11

**Authors:** Faliang Wang, Kiran Vij, Lin Li, Paarth Dodhiawala, Kian-Huat Lim, Jieya Shao

**Affiliations:** 1 Department of Surgical Oncology, The Children’s Hospital, Zhejiang University School of Medicine, National Clinical Research Center for Child Health, Hangzhou 310052, China; 21118179@zju.edu.cn; 2 Department of Medicine, Washington University School of Medicine, St. Louis, MO 63110, USA; kvij@wustl.edu (K.V.); lli@wustl.edu (L.L.); dodhiawalap@wustl.edu (P.D.); kian-huat.lim@wustl.edu (K.-H.L.); 3 Department of Pathology and Immunology, Washington University School of Medicine, St. Louis, MO 63110, USA; 4 Siteman Cancer Center, Washington University School of Medicine, St. Louis, MO 63110, USA

**Keywords:** DNA damage response, chemotherapy resistance, pancreatic ductal adenocarcinoma, chemotherapy predictive biomarker, synthetic lethality

## Abstract

**Simple Summary:**

Defining the mechanisms underlying the heterogeneous cancer cell response to DNA damage is important for our ability to tailor individualized strategies to improve chemotherapy efficacy. In this study, we focus on a central DNA repair regulator named VCP whose loss-of-function has been shown by prior studies to increase chemotherapy effects. However, since VCP participates in numerous cellular processes, its total expression level cannot differentiate between VCP-dependent vs. -independent tumors with specific regard to chemotherapy-induced DNA repair. Here, by examining cell lines and patient samples of pancreatic ductal adenocarcinoma (PDAC), we made two major observations. First, DNA damage-induced Ser^784^ phosphorylation is essential for DNA repair by VCP and drives chemo-resistance. Second, pSer^784^-VCP instead of total VCP protein predicts cellular reliance on VCP function during DNA repair and consequently chemo-sensitizing effects of VCP inhibition. Thus, pSer^784^-VCP may be a potential predictive biomarker and sensitizing target for cancer chemotherapy treatments.

**Abstract:**

Pancreatic ductal adenocarcinoma (PDAC) patients have a dismal prognosis due in large part to chemotherapy resistance. However, a small subset containing defects in the DNA damage response (DDR) pathways are chemotherapy-sensitive. Identifying intrinsic and therapeutically inducible DDR defects can improve precision and efficacy of chemotherapies for PDAC. DNA repair requires dynamic reorganization of chromatin-associated proteins, which is orchestrated by the AAA+ ATPase VCP. We recently discovered that the DDR function of VCP is selectively activated by Ser^784^ phosphorylation. In this paper, we show that pSer^784^-VCP but not total VCP levels in primary PDAC tumors negatively correlate with patient survival. In PDAC cell lines, different pSer^784^-VCP levels are induced by genotoxic chemotherapy agents and positively correlate with genome stability and cell survival. Causal effects of pSer^784^-VCP on DNA repair and cell survival were confirmed using VCP knockdown and functional rescue. Importantly, DNA damage-induced pSer^784^-VCP rather than total VCP levels in PDAC cell lines predict their chemotherapy response and chemo-sensitizing ability of selective VCP inhibitor NMS-873. Therefore, pSer^784^-VCP drives genotoxic chemotherapy resistance of PDAC, and can potentially be used as a predictive biomarker as well as a sensitizing target to enhance the chemotherapy response of PDAC.

## 1. Introduction

Pancreatic cancer remains the most lethal solid malignancy with >95% of the cases being ductal adenocarcinoma (PDAC). It is the third leading cause of cancer-related death in the USA with a 5-year survival rate of ~9% [1,2]. Unfortunately, neither immunotherapy nor targeting driver mutations have achieved significant clinical success so far. Combinatorial chemotherapy regimens such as FOLFIRINOX (leucovorin, 5-fluorouracil, oxaliplatin, irinotecan) and G/A (gemcitabine/paclitaxel) are the mainstay options for PDAC patients [3,4]. However, treatment response to these chemotherapy regimens is limited and not durable. The mechanisms behind heterogeneities in chemotherapy response remain poorly defined. Understanding them is important for two main reasons. First, it helps identify chemo-sensitive patients, and spare chemo-resistant patients from unnecessary toxicities. Second, it can provide mechanistic insights to guide the development of chemo-sensitizing strategies for the chemo-resistant cases.

Standard chemotherapy regimens for PDAC such as FOLFIRINOX and G/A mainly consist of genotoxins which trigger the DNA damage response (DDR). DDR involves numerous proteins that are spatiotemporally coordinated to repair genomic lesions and promote cellular survival. Consequently, DDR defects serve as the “Achilles heel” for genotoxic chemotherapies and increase treatment response [5]. This was best exemplified by the seminal findings that BRCA mutations in breast and ovarian cancers cause synthetic lethality with PARP inhibitors [6,7,8]. In December 2019, PARP inhibitor Olaparib became the first FDA-approved biomarker-driven targeted chemotherapy to treat germline BRCA-mutated metastatic PDAC after initial treatment with FOLFIRINOX. Thus, identifying naturally occurring, as well as therapeutically inducing, DDR defects holds great promise for improving chemotherapy response of PDAC patients [9].

DNA repair requires dynamic protein reorganization on chromatin [10,11,12], orchestrated by the AAA+ ATPase valosin-containing protein (VCP). VCP liberates K48-polyubiquitinated proteins from chromatin, membrane organelles (e.g., ER) and macromolecular complexes (e.g., ribosome) to facilitate their timely turnover [13,14,15]. The critical role of VCP in DNA repair was established by many studies primarily utilizing genetic loss-of-function and chemical inhibitory approaches [16,17,18,19,20,21]. However, given that VCP exerts pleiotropic effects on global proteostasis throughout the cell, its total expression levels are not expected to accurately inform its DDR-specific activity. VCP is phosphorylated at Ser^784^ in its C-terminal tail by the PIKK kinases (ATM/ATR/DNA-PK) in response to different genotoxic insults [22,23,24]. However, the functional importance of Ser^784^ phosphorylation remained unknown until our recent study [25,26]. Using an isogenic knockdown and rescue approach, we demonstrated that Ser^784^ phosphorylation of VCP is essential for chromatin-associated protein degradation, DNA repair, checkpoint signaling, and cellular survival in response to diverse genotoxic chemotherapeutic treatments. However, Ser^784^ phosphorylation does not affect VCP function during ER-associated protein degradation and cytoplasmic protein clearance. Importantly, by staining multiple primary breast tumor microarrays using custom-generated antibodies, we observed significant association between nuclear pSer^784^-VCP levels and poor patient survival in a chemotherapy-dependent fashion. These data collectively suggest that pSer^784^-VCP is an essential DDR regulator which may drive resistance of a broad range of cancer types to genotoxic chemotherapies. They also imply that pSer^784^-VCP may be a novel chemotherapy-sensitizing target. Nonetheless, the clinical relevance of pSer^784^-VCP for chemotherapy resistance of PDAC has not been investigated. In this paper, we provide clinical and experimental evidence that pSer^784^-VCP drives chemotherapy resistance of PDAC and its intratumor levels predict chemotherapy efficacy as well as chemo-sensitizing effect of pharmacologic VCP inhibitors. 

## 2. Materials and Methods

### 2.1. Cell Lines

All cell lines used in this study were purchased from ATCC with the exception of patient-derived Pa01C as previously described [27,28]. All cell lines including PANC-1, MIA PaCa-2, Capan-1, Pa01C, and HEK293T were mycoplasma-tested annually and cultured in high glucose DMEM supplemented with 10% fetal bovine serum and 50 mg/mL gentamicin. Lentiviruses were produced by transient transfection of HEK293T cells as previously described [25,29,30,31].

### 2.2. DNA and shRNA Constructs

YFP-VCP (WT, S784A, or S784D) containing silent mutations conferring resistance to two different shRNAs targeting human VCP were generated in the lentiviral pFLRu-NYFP-FH vector by subcloning. Briefly, RNAi-resistant VCP (WT, S784A, or S784D) cDNAs were digested from the previously described VCP-GFP constructs using BglII (5’) and AgeI (3’) and inserted into the BamHI and AgeI sites of the pFLRu-NYFP-FH vector [25,30,32]. Two distinct shRNAs targeting human VCP were described previously [25].

### 2.3. Human PDAC Tissue Microarray (TMA)

The PDAC TMA consisting of totally 165 separate PDAC tumors (each with duplicate cores) has been previously described [28,33]. In this study, 24 samples that were either depleted or lacked sufficient number of tumor cells for accurate scoring were excluded.

### 2.4. Immunofluorescence Staining and Image Acquisition

PDAC cell lines were treated with DNA damaging agent as indicated in the figure legends and subjected to immunofluorescence staining for pSer^784^-VCP and γH2AX as previously described. Briefly, cells were grown in 96-well plates, treated, and detergent extracted for 8 min, fixed by 4% paraformaldehyde for 15 min, washed by PBS/0.1% Triton X-100, blocked with 5% normal goat serum for 60 min, and incubated with primary antibodies (1:1000; custom-made mouse anti-pSer^784^-VCP and rabbit anti-γH2AX, Cell Signaling #9718) overnight at 4 °C. Cells were washed with PBS/0.1% Triton X-100, incubated with Alexa 488 or 594-conjugated secondary antibodies (Invitrogen, Waltham, MA, USA) for 2 h at room temperature, washed with PBS/0.1% Triton X-100, and counterstained with DAPI. Fluorescence images were acquired on an inverted Olympus IX70 microscope equipped with CellSens software as previously described [25,30,31].

### 2.5. Immunohistochemistry and Scoring

IHC for total VCP and pSer^784^-VCP were performed using standard protocol as previously described. Briefly, 4 μm PDAC tissue microarray sections were de-paraffinized, rehydrated, and subjected to antigen retrieval by heating for 10 min in Citrate buffer, pH 6.0 for total VCP and Tris buffer, pH 9.0 for pSer^784^-VCP as previously described [25]. Tissues were subsequently quenched by hydrogen peroxide, washed, blocked, and incubated with primary antibodies (Santa Cruz mouse monoclonal anti-VCP at 1:2000, sc-136273; custom mouse monoclonal anti-pSer^784^-VCP at 1:1000) at 4 °C overnight. Tissues were washed, incubated for 2 h at room temperature with HRP-conjugated secondary antibody (SignalStain Boost IHC Detection Reagent Mouse, #8125, Cell Signaling, Danvers, MA, USA), washed, and developed using the ImmPACT DAB kit (Vectorlabs, SK-4105, Burlingame, CA, USA). They were subsequently counterstained with hematoxylin, dehydrated, and mounted. Bright field images were taken on an upright BX51 fluorescence microscope using the CellSens software. All images were taken at the same exposure time and settings. For the quantification of IHC signals, Image-Pro Plus 6.0 (IPP 6.0, Media Cybernetics, Inc., Rockville, MD, USA) was used as described previously [34,35,36]. Briefly, three non-overlapping fields of each IHC image were randomly selected and the integrated optical density (IOD) of each area of interest was quantified. Normalized density (AOD/area) was calculated and averaged among the six areas of interest for each tumor sample (3 areas of interest within each duplicate core) to represent the expression levels of protein markers (pSer^784^-VCP or total VCP).

### 2.6. Comet Assay

The experiment was performed under alkaline conditions as previously described using the Single Cell Gel Electrophoresis Assay kit from Trevigen (cat # 4250-050-K, Gaithersburg, MD, USA). Fluorescent images were taken using the Cellsens software on a 10× objective of an Olympus IX70 microscope and analyzed by ImageJ (ImageJ 1.8.0, Bethesda, MD, USA) under the OpenComet plugin (http://cometbio.org/, accessed on 17 August 2021) [37]. All images were taken with the same exposure time and magnification. Data were expressed by the tail DNA percentage (tail intensity out of total DNA intensity).

### 2.7. Cellular Viability and Colony Formation Assays

For the cell viability assay, PDAC cells were plated in triplicates in 96-well plates at 2000-cells-per-well density and allowed to adhere overnight. Cells were treated for 4 days with vehicle (DMSO) or 2-fold increasing concentrations of SN38 (1.56 nM–200 nM), Etoposide (39 nM–5 μM), NMS-873 (7.8 nM–1 μM) alone or in combination. Ratios of NMS-873/SN38 and NMS-873/Etoposide in concentration were kept fixed at 5:1 and 1:5, respectively, for all combinations. Viable cells were quantified by Alamar blue, and dose–response curves were generated using Graphpad Prism (GraphPad Prism Software 8.0.1, San Diego, CA, USA) based on relative cell survival (drug relative to vehicle). For colony formation assay, PDAC cells were plated in triplicates in 24-well plates at a 400-cells-per-well density, allowed to adhere overnight, treated with SN38 or Etoposide for 16 hours, and grown in drug-free media for 14 days. End-point viable cells were quantified by Alamar blue and colonies were visualized by crystal violet staining as previously described [25,30,31].

### 2.8. Western Blot

Cell lysates were prepared using RIPA buffer, and soluble and insoluble fractions were analyzed as previously described [25,30].

### 2.9. Statistical Analysis

For comet assays, significance in difference was calculated based on unpaired *t*-tests using Graphpad Prism. For clonogenic assays, unpaired *t*-test (single drug doses) and one-way ANOVA and Dunnett’s multiple comparisons (dose-dependent curves) were used to calculate statistical significance. For significance in difference in overall survival of PDAC patients, Kaplan–Meier curves were analyzed based on Log-rank and Wilcoxon tests using Graphpad Prism. For multivariate analyses, Cox proportional hazards models were used to assess the hazard ratio (HR) of different prognostic covariates with survival using R software (version 3.5.3, R Foundation for Statistical Computing, Vienna, Austria). Analyses including multiple comparisons were adjusted for multiple testing using the Benjamini–Hochberg FDR method. *p* < 0.05 was considered as statistically significant. The estat phtest command in Stata 12.0 was used to determine whether the proportional hazards assumption was violated (*p* > 0.05 indicating no violation) for each predictor as well as to carry out the global test. 

## 3. Results

### 3.1. DNA Damage-Induced pSer^784^-VCP Levels Vary among PDAC Cell Lines

**Figure 1:**
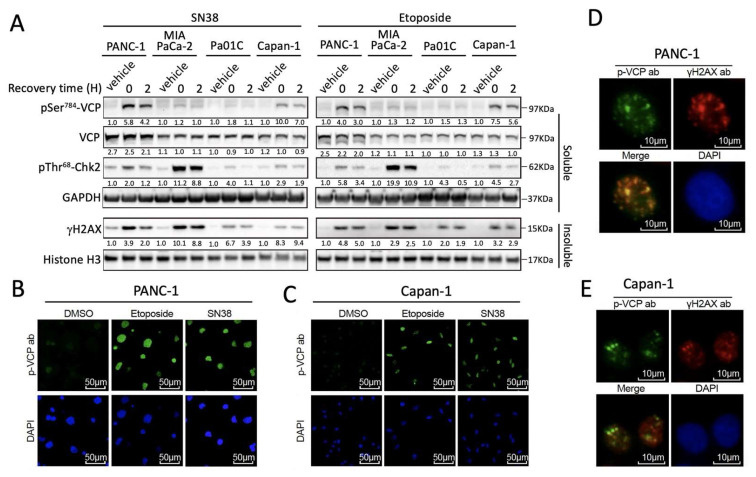
DNA damage-induced pSer^784^-VCP levels vary among PDAC cell lines. (**A**) Four distinct PDAC cell lines were treated identically with vehicle control DMSO, 2 µM SN38, or 50 µM Etoposide for 4 h, recovered or not for 2 h, and lysed with RIPA buffer. Soluble and insoluble fractions were analyzed by Western blot for the indicated proteins. Band intensities were quantified by densitometry and normalized over GAPDH (for soluble proteins) and histone H3 (for insoluble proteins). For pSer^784^-VCP, pThr^68^-Chk2, and γH2AX, relative changes in their levels upon drug treatments were separately calculated for each cell line (vehicle levels arbitrarily set at 1). For total VCP, its level was compared across all four cell lines treated or not with drugs and expressed at relative values. The uncropped western blot figure was presented in Figure S3. (**B**,**C**) PANC-1 and Capan-1 cells were treated with DMSO, 2 µM SN38, or 50 µM Etoposide for 4 h, recovered by 2 h, detergent-extracted, fixed, and subjected to immunofluorescence staining for pSer^784^-VCP. Nuclei were counterstained by DAPI. (**D**,**E**) SN38-treated PANC-1 and Capan-1 cells were subjected to double immunofluorescence staining for pSer^784^-VCP and γH2AX, and counterstained with DAPI. Merged images show nuclear foci positive for both pSer^784^-VCP and γH2AX. All results have been independently confirmed at least three times.

Consistent with the fact that VCP is highly abundant and ubiquitously expressed [13], we detected similar expression levels of VCP protein based on Western blot across three different established PDAC cell lines (MIA PaCa-2, PANC-1, and Capan-1), and a patient-derived PDAC cell line Pa01C [27,28]. Interestingly, when treated with SN38, the active metabolite of irinotecan, which is a DNA topoisomerase I inhibitor used to treat PDAC as part of FOLFIRINOX [3,4], the cell lines showed distinct levels of pSer^784^-VCP induction. While PANC-1 and Capan-1 cells showed robust pSer^784^-VCP induction, little pSer^784^-VCP was found in MIA PaCa-2 and Pa01C cells (Figure 1A). The levels of pSer^784^-VCP do not correlate with those of other PIKK substrates pSer^345^-Chk1 [38,39,40] or γH2AX [41,42], arguing against the possibility that the differential pSer^784^-VCP induction is simply due to cell-line-dependent difference in DDR signaling. Etoposide, a clinical inhibitor of DNA topoisomerase II and robust inducer of DNA double-strand breaks [43], triggers the same pattern of pSer^784^-VCP induction as SN38 in the four PDAC cell lines (Figure 1A), demonstrating that it is not a drug-specific phenomenon. Consistent with our prior findings using non-PDAC cell lines (HeLa and U2OS) [25], pSer^784^-VCP is detected in the nuclei of both PANC-1 and Capan-1 cell lines (Figure 1B,C) and within γH2AX-positive DNA damage foci (Figure 1D,E) upon drug treatment. Therefore, pSer^784^-VCP is differentially induced by DNA damage in different cellular contexts despite similar expression levels of total VCP protein.

### 3.2. pSer^784^-VCP Levels Predict the Chemotherapy Effect on Genome Integrity and Sensitizing Effect of VCP Inhibitor in PDAC Cell Lines

**Figure 2:**
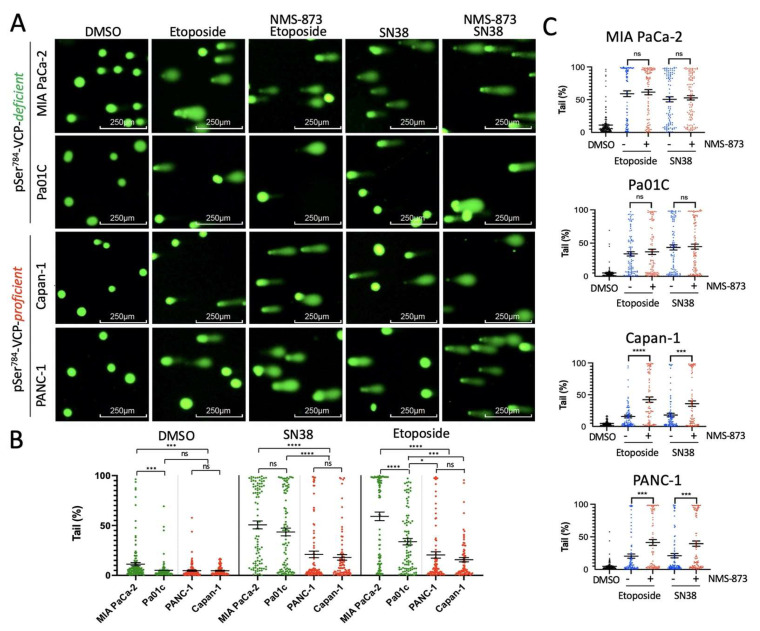
pSer^784^-VCP levels predict chemotherapy effect on genome integrity and sensitizing effect of VCP inhibitor in PDAC cell lines. pSer^784^-VCP-deficient MIA PaCa-2 and Pa01C vs. pSer^784^-VCP-proficient Capan-1 and PANC-1 cells were treated for 2 h with DMSO, 50 µM Etoposide, or 2 µM SN38 in the absence or presence of 10 µM VCP inhibitor NMS-873. Cells were subjected to the comet assay under alkaline conditions, and comet tail percentages were calculated. (**A**) Representative comet images under each experimental condition. (**B**) Quantification of comet tail percentages of all four cell lines in response to DMSO, SN38, or Etoposide treatments. *p* values were based on one-way ANOVA (within each treatment group) and Tukey’s multiple comparisons test. (**C**) Same comet tail data were represented separately for each cell line with and without NMS-873 co-treatment. *p* values were based on unpaired student *t*-test. A total of 100–200 cells were analyzed per experimental condition. Similar results were independently observed three times. Error bars represented standard error of the mean (SEM). * *p* < 0.05; *** *p* < 0.001; **** *p* < 0.0001.

Given the various extents of pSer^784^-VCP induction by genotoxic insults in the different PDAC cell lines, we asked whether this is sufficient to influence the repair efficiency of chemotherapy-induced DNA damage. To test this, we treated all four PDAC cell lines with DMSO vehicle, SN38, or Etoposide for 2 h and measured DNA breaks using the comet assay. We found that both drugs cause significantly more DNA damage, indicated by higher percentages of comet tails per nucleus, in MIA PaCa-2 and Pa01C than PANC-1 and Capan-1 cells (Figure 2A,B). The levels of DNA damage in the PDAC cell lines correlate closely and inversely with their abilities to induce pSer^784^-VCP. This is consistent with the notion that pSer^784^-VCP plays a dominantly important role in DNA repair that is sufficient to influence genome stability. Intrigued by this finding, we next asked whether deactivating VCP in PDAC cell lines containing high levels of pSer^784^-VCP would increase chemotherapy-induced DNA damage due to the loss of the genome-protective effect of pSer^784^-VCP. To test this, we co-treated the four PDAC cell lines with SN38 or Etoposide together with NMS-873, a highly specific allosteric VCP inhibitor targeting its ATPase activity [44,45]. Compared to SN38 or Etoposide alone, simultaneous treatment with NMS-873 significantly increased their DNA damaging effects in PANC-1 and Capan-1 cells in which pSer^784^-VCP can be robustly induced (Figure 2C). In contrast, co-treatment with NMS-873 in MIA PaCa-2 and Pa01C cells showed no further DNA damaging effect compared to SN38 and Etoposide alone, consistent with their lack of pSer^784^-VCP induction. These results have two major implications. First, pSer^784^-VCP levels predict chemotherapy-induced DNA damage. Second, pSer^784^-VCP levels signify cellular reliance on VCP activity for DNA repair and predict chemotherapy-sensitizing effect of VCP inhibitor.

### 3.3. pSer^784^-VCP Levels Predict Chemotherapy Effect on Cell Survival and Sensitizing Effect of VCP Inhibitor in PDAC Cell Lines

**Figure 3:**
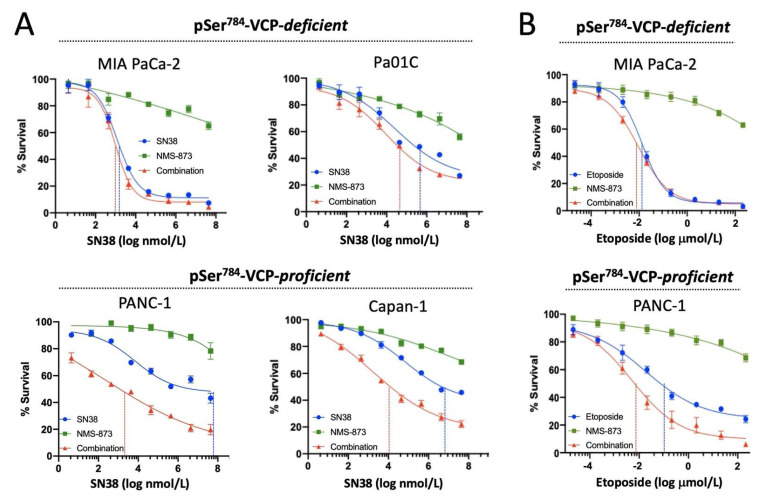
pSer^784^-VCP levels predict chemotherapy effect on cell survival and sensitizing effect of VCP inhibitor in PDAC cell lines. (**A**) pSer^784^-VCP-deficient MIA PaCa-2 and Pa01C vs. pSer^784^-VCP-proficient Capan-1 and PANC-1 cells were treated for 5 days with DMSO control or 2-fold increasing concentrations of SN38 (1.56 nM–200 nM) and NMS-873 (7.8 nM–1 μM) individually or in combination at a fixed 1:5 ratio. End-point cell viability was measured by Alamar blue assay, and relative survival of drug-treated vs. vehicle-treated cells was calculated and plotted against SN38 concentrations in dose–response curves. NMS-873 concentrations were not shown in the plots for simplicity. Similar results were observed within three independent experiments. Shown are data from one representative experiment. Data are mean ± SEM. Dashed lines in each dose–response curve indicated the drug concentrations at which 50% of cell survival relative to vehicle control was observed. They were color-coded to indicate whether they reflected single drug treatments (blue) or combination treatments (red). (**B**) MIA PaCa-2 and PANC-1 cells were treated for 5 days with Etoposide (39 nM–5 μM) and NMS-873 (7.8 nM–1 μM) individually or in combination at a fixed 5:1 ratio. Relative survival was calculated and dose–response curves were generated as described in A. Similar results were observed within three independent experiments. Shown are data from one representative experiment.

Next, we asked whether the different levels of pSer^784^-VCP induction and DNA damage in these PDAC cell lines can influence their susceptibility to chemotherapy. To do this, we treated the four PDAC cell lines with DMSO or twofold increasing concentrations of SN38 (1.56 nM–200 nM) for 5 days in 96-well plates, and quantified end-point cell viability using the Alamar blue assay. Dose–response curves showed that MIA PaCa-2 is the most sensitive cell line followed sequentially by Pa01C, Capan-1, and PANC-1, based on the concentrations of SN38 at which 50% of cell survival relative to vehicle control was observed (Figure 3A). The relative sensitivities of the PDAC cell lines to SN38 inversely correlate with their drug-induced pSer^784^-VCP levels (Figure 1A) and parallel to the amounts of drug-induced DNA damage (Figure 2), further supporting the idea that pSer^784^-VCP is a critically important DDR regulator capable of driving chemotherapy resistance. Next, we tested whether VCP inhibition can preferentially increase the cytotoxic effects of chemotherapy treatment in PDAC cell lines with the robust induction of pSer^784^-VCP upon DNA damage. Indeed, compared to SN38 alone, combination treatment of SN38 plus NMS-873 caused further decrease of cell viability specifically in pSer^784^-VCP-proficient PANC-1 and Capan-1 cells but not in pSer^784^-VCP-deficient MIA PaCa-2 and Pa01C cells (Figure 3A). Notably, NMS-873 alone mildly decreased cell viability with a similar magnitude across all four PDAC cell lines (Figure 3A). To determine whether this may be a drug-specific phenomenon, we selected MIA PaCa-2 as a highly SN38-sensitive and pSer^784^-VCP-deficient PDAC cell line and PANC-1 as a highly SN38-resistant and pSer^784^-VCP-proficient PDAC cell line and performed dose–response survival analysis using Etoposide and NMS-873 alone or in combination. Similar to SN38, Etoposide is more effective at decreasing the survival of MIA PaCa-2 than PANC-1 cells (Figure 3B). Thus, not only can pSer^784^-VCP significantly influence DNA repair and cellular survival upon chemotherapy treatments, but it also appears to be a reliable biomarker predicting cellular dependence on VCP function during DDR and consequently chemo-sensitizing effect of VCP inhibition.

### 3.4. pSer^784^-VCP Protects PDAC Cell Lines from Chemotherapy-Induced DNA Damage and Cell Death

**Figure 4:**
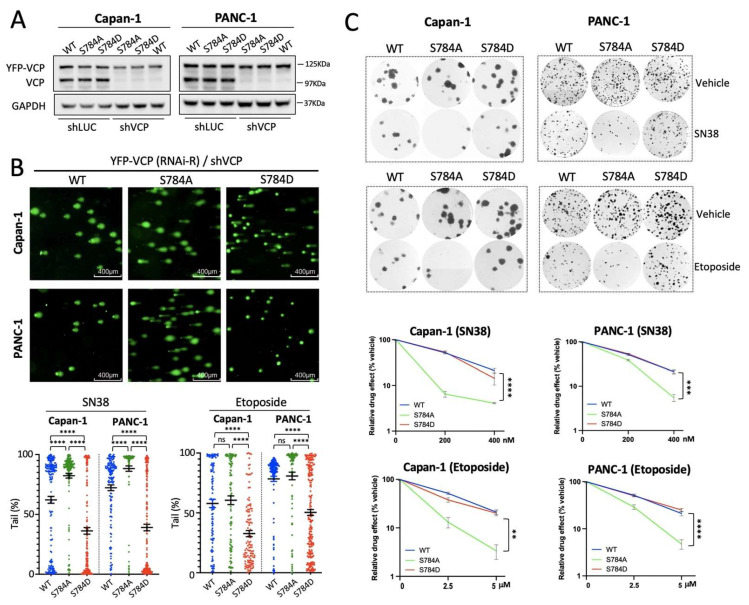
pSer^784^-VCP protects PDAC cell lines from chemotherapy-induced DNA damage and cell death. (**A**) Capan-1 and PANC-1 cells were infected first with lentiviruses expressing RNAi-resistant YFP-VCP (WT, S784A, or S784D) followed by lentiviral infection with two combined shRNAs targeting human VCP. Complete knockdown of endogenous VCP but not RNAi-resistant YFP-VCP in both cell lines were shown by Western blot. The uncropped western blot figure was presented in Figure S3. (**B**) Capan-1 and PANC-1 cells whose endogenous VCP was replaced by RNAi-resistant YFP-VCP (WT, S784A, or S784D) via knockdown/rescue were treated with SN38 (2 μM) and Etoposide (50 μM) for 2 h and subjected to the comet assay to quantify DNA breaks. Representative images of SN38-treated cells were shown. Comet tail percentages of 100–200 cells per condition were calculated. Data are mean ± SEM of one representative experiment, and similar results were confirmed in three independent experiments. *p* values were based on unpaired *t*-test. (**C**) VCP knockdown and rescue Capan-1 and PANC-1 cells (as in **B**) were treated with DMSO, SN38 (200 nM and 400 nM), and Etoposide (2.5 μM and 5 μM) for 16 h and subsequently cultured in drug-free media for 14 days in colony formation assays. Viable cells were measured by Alamar blue followed by crystal violet staining. Representative images of cell colonies were shown. Relative survival of drug-treated vs. vehicle-treated cells based on Alamar blue values were plotted. Data are mean ± SEM. Similar results were confirmed by three independent experiments. *p* values were based on one-way ANOVA and Dunnett’s multiple comparisons of WT or S784D vs. S784A at the highest drug concentrations. **, *p* < 0.01; ***, *p* < 0.001; ****, *p* < 0.0001.

To further confirm that chemotherapy-induced pSer^784^-VCP causally influences rather than simply correlates with the DNA repair efficiency and survival of PDAC cells, we took an isogenic approach using the pSer^784^-VCP-proficient PANC-1 and Capan-1 cells. Using a previously described genetic knockdown and rescue strategy [25], we replaced the endogenous VCP in both cell lines with YFP-tagged RNAi-resistant VCP lacking (wild type) or containing either a S784A or S784D mutation to mimic the unphosphorylated or Ser^784^-phosphorylated VCP. Since Ser^784^ phosphorylation is not required for VCP function in the absence of DNA damage, this is an ideal approach to specifically assess the DDR-relevant activity of pSer^784^-VCP upon chemotherapy treatments without the potential indirect effects of basal cell viability differences. Western blot analysis showed that similar levels of YFP-VCP (WT, S784A, and S784D) were expressed relative to endogenous VCP in both cell lines, and shVCP completely silenced endogenous VCP but not exogenous YFP-VCP (Figure 4A). First, we examined chemotherapy-induced DNA damage using the comet assay. Compared to YFP-VCP(WT)-rescued PANC-1 and Capan-1 cells, those rescued with YFP-VCP(S784A) showed significantly more SN38-induced DNA breaks while those rescued with YFP-VCP(S784D) showed significantly less (Figure 4B). As for Etoposide treatment, YFP-VCP(S784D)-rescued cells showed significantly fewer DNA breaks than those rescued with YFP-VCP(WT) or YFP-VCP(S784A). Though not statistically significant, more Etoposide-induced DNA breaks could be observed in YFP-VCP(S784A)-rescued cells compared to those rescued with YFP-VCP(WT) (Figure 4B). Next, we examined cell survival upon chemotherapy treatment using the colony formation assay. Consistent with the importance of VCP for DDR, VCP knockdown in parental PANC-1 cells increased their sensitivity to SN38 and Etoposide treatments (Figure S1). Both VCP knockdown PANC-1 and Capan-1 cells rescued by YFP-VCP(WT) and YFP-VCP(S784D) exhibited significantly higher resistance to SN38 and Etoposide than those rescued by YFP-VCP(S784A) (Figure 4C). These isogenic data confirmed that pSer^784^-VCP promotes DNA repair and survival of PDAC cells upon genotoxic chemotherapies and causally drives treatment resistance.

### 3.5. pSer^784^-VCP Levels Significantly Associate with Poor Survival of PDAC Patients

**Figure 5:**
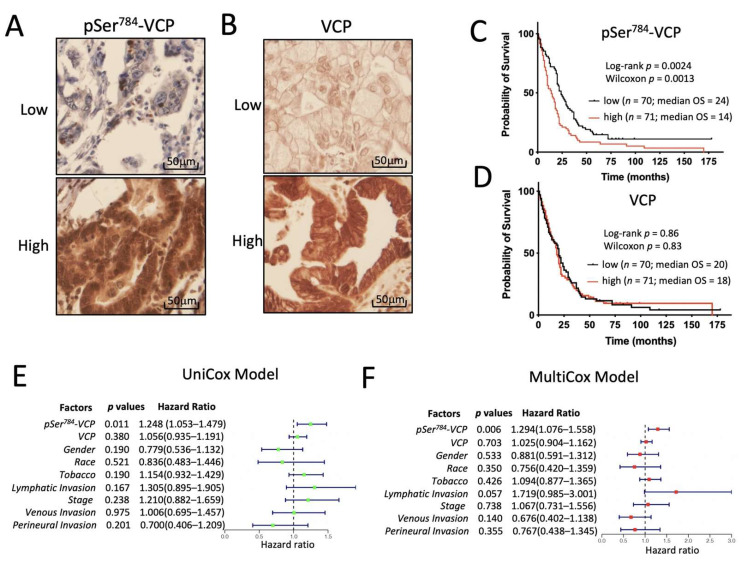
pSer^784^-VCP levels significantly associate with poor survival of PDAC patients. (**A**,**B**) Representative IHC images of PDAC tissues containing high or low intratumor levels of pSer^784^-VCP (**A**) and total VCP (**B**). (**C**,**D**) Kaplan–Meier analysis of PDAC patients showing statistically significant association between high levels of pSer^784^-VCP (**C**) but not total VCP (**D**) with poor overall survival. IHC signals of pSer^784^-VCP and total VCP were scored blindly and unbiasedly using Image-Pro Plus 6.0 software. A total of 141 cases with sufficient scorable tumor cells in the cohort were dichotomized into two sub-groups containing high vs. low levels of total VCP or pSer^784^-VCP. Kaplan–Meier curves were generated using Graphpad Prism. *p* values were based on Log-rank and Wilcoxon tests as indicated in the graphs. Median overall survival months of the stratified sub-groups were also shown. (**E**,**F**) Univariant (**E**) and multivariant (**F**) Cox regression models were built to estimate Hazard Ratios (HR) for pSer^784^-VCP and total VCP based on their intratumor IHC levels, along with other available clinical variables and risk factors.

To seek clinical evidence for the functional importance of pSer^784^-VCP for DNA repair and PDAC cell survival upon chemotherapy treatments, we examined the intratumoral levels of pSer^784^-VCP in patient tumor samples of a clinically annotated PDAC tissue microarray (TMA) by immunohistochemistry (IHC) [33,46]. Nearly all patients in this cohort received standard post-surgery chemotherapy treatments consisting of genotoxic agents such as gemcitabine, 5FU, and cisplatin. We stained the PDAC TMA for pSer^784^-VCP and total VCP and quantified the IHC signals using the Image-Pro Plus 6.0 software. Varying levels of total VCP and pSer^784^-VCP were detected across different PDAC tissues (Figure 5A,B). First, we performed Kaplan–Meier analysis of overall patient survival by dichotomizing the dataset evenly into high vs. low pSer^784^-VCP or total VCP sub-groups based on their median IHC levels. This revealed that high pSer^784^-VCP levels are significantly associated with worse overall survival (OS) of PDAC patients based on both Log-rank and Wilconxin tests (Figure 5C). In contrast, total VCP levels are not associated with patient survival (Figure 5D). Next, we built Cox proportional hazards regression models to estimate the hazard ratios (HR) for both pSer^784^-VCP and total VCP. Results from the univariate model analysis showed that pSer^784^-VCP (*p* = 0.011; HR = 1.248; 95% confidence interval = 1.053 to 1.479) but not total VCP (*p* = 0.38; HR = 1.056; 95% CI = 0.935 to 1.191) is a significant poor prognostic factor associated with OS. Interestingly, none of the available clinical variables and risk factors including the cancer stage, gender, race, tobacco use, or cancer cell invasion showed statistically significant prognostic values (Figure 5E). To take into consideration potential confounders, a multivariate Cox regression model was built to assess the association between pSer^784^-VCP and OS, including the covariates of total VCP and the same set of clinical variables and risk factors as described above. This resulted in a statistically significant adjusted HR of pSer^784^-VCP at 1.294 (*p* = 0.006; 95% CI = 1.076 to 1.558), meaning that the probability of OS among patients with high levels of pSer^784^-VCP was 29% lower than among those with low levels of pSer^784^-VCP (Figure 5F). This was further confirmed by a proportional hazard assumption test, which found no evidence of violation (Figure S2 and Table S1). Therefore, pSer^784^-VCP independently prognosticates poor survival of PDAC patients who routinely receive standard genotoxic chemotherapies.

## 4. Discussion

Despite the well-accepted notion that DNA damage response (DDR) defects render cancer cells sensitive to genotoxic chemotherapies, we do not yet know how to precisely identify such defects in the tumors to stratify patients for effective treatments due to the shortage of reliable predictive biomarkers. Part of the challenge lies in the fact that DDR is an adaptive cellular process which is only robustly induced when there is sufficient amount of DNA damage [11,12]. Additionally, post-translational modification (e.g., phosphorylation) plays a crucially important role in turning on and off many DNA repair proteins and signaling molecules [10,11,23,24]. As such, although DNA sequencing can help identify a small subset of tumors carrying loss-of-function mutations in key DDR factors and predict their sensitivity to specific chemotherapies such as the platinums or topoisomerase inhibitors, other tumors that are DDR-deficient on the functional level, especially due to protein post-translational modifications, cannot be genetically identified. Recognizing the dynamic and protein-centric nature of DDR, recent studies have provided promising proof-of-principle evidence demonstrating the clinical utility of functional protein DDR markers. The most notable example is the independent validation of treatment-induced nuclear Rad51 foci as a functional marker of homologous recombination (HR) within clinical samples of breast and ovarian cancer and its ability to predict patient response to PARP inhibitors and platinum drugs [47,48,49,50,51,52,53]. 

In this study, we built on our recent identification of pSer^784^-VCP as an important DDR regulator and present new evidence that it is functionally required for DNA repair and cellular survival of chemotherapy-treated PDAC. There are two important implications of our work. First, our cell line and patient data suggested that pSer^784^-VCP instead of total VCP levels predict PDAC response to genotoxic chemotherapies. This is conceptually consistent with the pleiotropic effects of VCP on global proteostasis [13,14,15,54] and the specific activating effect of Ser^784^ phosphorylation on DDR function of VCP [25,26]. As with our prior study using surgical breast tumor samples [25], the PDAC TMA used in this study was constructed using tumor samples collected at surgery before adjuvant chemotherapies given to nearly all patients. Therefore, the pSer^784^-VCP signals detected in the tumors reflect its baseline levels triggered by tumor-intrinsic genome instability and DNA damage and not by chemotherapy or other therapeutic interventions. Despite the fact that baseline pSer^784^-VCP levels significantly correlate with poor patient survival, it is easily conceivable that many DDR-proficient and chemotherapy-resistant tumors do not harbor sufficient baseline DNA damage to induce pSer^784^-VCP. Therefore, our current data most likely underestimated the true chemotherapy-predictive ability of pSer^784^-VCP which will require future validation using tumor samples that are undergoing chemotherapy treatments. In addition, given that VCP orchestrates multiple DNA repair pathways by enabling dynamic chromatin protein reorganization [16,55,56,57,58], and prior and current findings from us and others that Ser^784^ phosphorylation can be induced by a wide range of DNA damaging agents [22,23,24,25], pSer^784^-VCP can potentially dictate the response of different cancer types to diverse genotoxic chemotherapeutic agents including PARP inhibitors which were recently approved to treat germline BRCA-mutated metastatic PDAC. Interestingly, recent studies suggested that DDR defects can increase tumor mutation and neoantigen load as well as cytosolic DNA triggering innate immune response through the cyclic GMP-AMP synthase (cGAS) and STING pathway [59,60]. As such, DDR defects are potential predictors and sensitizers of the effects of immune checkpoint blockage. Given the urgent need to improve the PDAC patient response to immunotherapies and the critical role of pSer^784^-VCP in DDR, future studies are warranted to also investigate the impact of pSer^784^-VCP on tumor microenvironment and its clinical implication for cancer immunotherapies. 

The second important implication of our work is that the level of pSer^784^-VCP, instead of total VCP, signifies cellular reliance on VCP function during DDR and thereby predicts the chemotherapy-sensitizing effect of VCP inhibition. As a targetable enzyme instrumental for protein quality control, VCP has drawn considerable therapeutic attention in many disease areas including cancer. Multiple pharmacological inhibitors targeting the ATPase activity of VCP have been generated including the allosteric inhibitor NMS-873 used in this study and demonstrated promising proof-of-concept anticancer effects in different preclinical models [44,45,61,62,63]. Excitingly, a highly potent and selective ATP-competitive VCP inhibitor named CB-5339 is currently being evaluated in phase I clinical trials for acute myeloid leukemia (AML) and myelodysplastic syndromes (MDS) and is anticipated to enter clinical testing for solid tumors as well. Most recently, it was reported that CB-5339 is highly effective at inhibiting in vitro and in vivo growth and survival of AML cell lines and mouse xenograft models as monotherapy [64]. Interestingly, the authors also showed that CB-5339 synergizes with standard chemotherapies consisting of genotoxic anthracycline and cytarabine, supporting the general idea that VCP inhibition can be chemo-sensitizing. Not surprisingly, neither total VCP nor phospho-ATM and γH2AX levels correlate with the anticancer effects of CB-5339 [64]. Genetic profiling revealed an association between co-occurrence of RAS oncogenic activation and TP53 deficiency in AML cell lines and reduced the sensitivity to VCP inhibition. Nonetheless, since the analysis was conducted for the mono-therapeutic effect of VCP inhibitor, the potential ability of RAS/TP53 genetic alterations to predict the chemo-sensitizing effect of VCP inhibition remains unclear. These data support the general notion that VCP inhibition is a promising strategy to overcome resistance of various cancer types to a broad range of genotoxic chemotherapies but also underscore the need to find reliable predictive biomarkers in order to effectively harness its clinical potential. Therefore, by demonstrating the ability of pSer^784^-VCP to predict both the chemotherapy response and chemo-sensitizing effect of VCP inhibition in the context of PDAC, our study provided novel and important proof-of-concept data which directly addressed this urgent need and set the stage for future experiments to further validate and ultimately translate pSer^784^-VCP into the clinic to improve the therapeutic outcome of this deadly disease.

## 5. Conclusions

In conclusion, we provided experimental and clinical evidence that pSer^784^-VCP drives PDAC resistance to genotoxic chemotherapies, consistent with the fact that Ser^784^ phosphorylation robustly activates VCP, specifically in the context of DNA damage response. Our data suggest that intratumor levels of pSer^784^-VCP rather than total VCP protein accurately reflect cellular reliance on the VCP function during DNA damage response and may be used to predict both the chemotherapy response and chemo-sensitizing effect of pharmacological VCP inhibitors. Given the ubiquitously important role of VCP in DNA damage response across various tissue types, our findings likely have far-reaching relevance for other cancer types in addition to PDAC.

## Data Availability

The data presented in this study are available on request from the corresponding author.

## Supplementary Materials

The following are available online at https://www.mdpi.com/article/10.3390/cancers13205076/s1, Figure S1: VCP knockdown sensitizes PANC-1 cells to genotoxic chemotherapies, Figure S2: Visual assessment of proportional hazard assumption for pSer^784^-VCP, Figure S3: raw Western bot data, Table S1: Proportional hazard assumption analysis for pSer^784^-VCP.
