# Supplementary material for: Phospho-Ser784-VCP Drives Resistance of Pancreatic Ductal Adenocarcinoma to Genotoxic Chemotherapies and Predicts the Chemo-Sensitizing Effect of VCP Inhibitor
Source: Cancers (Basel). 2021 Oct 11;13(20):5076. doi: 10.3390/cancers13205076 (PMC8534018; doi:10.3390/cancers13205076)
Supplement: Supplementary file 1 [file cancers-13-05076-s001.zip › cancers-1365385-supplementary.pdf]

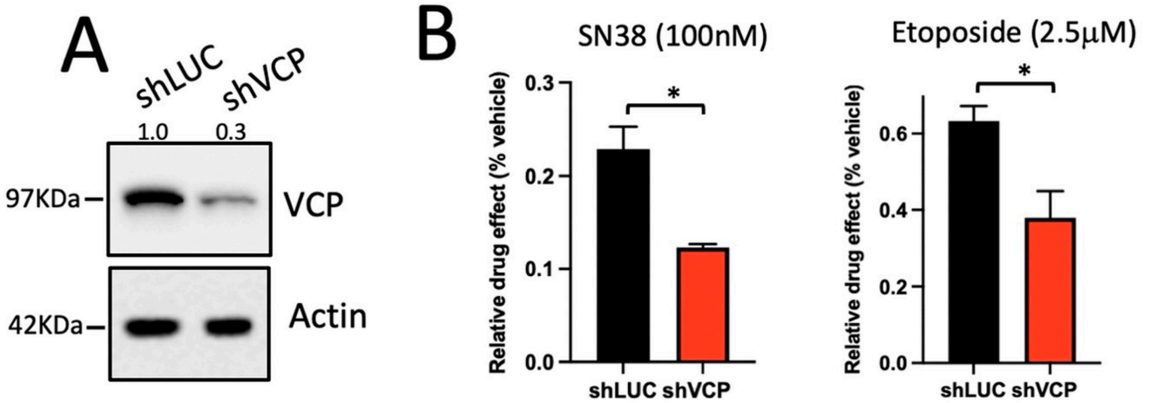


**Figure S1.** VCP knockdown sensitizes PANC-1 cells to genotoxic chemotherapies, related to Figure 4. (**A**) Western blot showing VCP knockdown (combined shVCP #1 and #2), controlled by shLUC, in PANC-1 cells. 70% of total VCP was knocked down based on densitometry analysis. The uncropped Western blot figure was presented in Figure S3. (**B**) Clonogenic assays using shLUC or shVCP-infected PANC-1 cells treated with SN38 or Etoposide. Data are mean ± SEM. P values are based unpaired t-test.

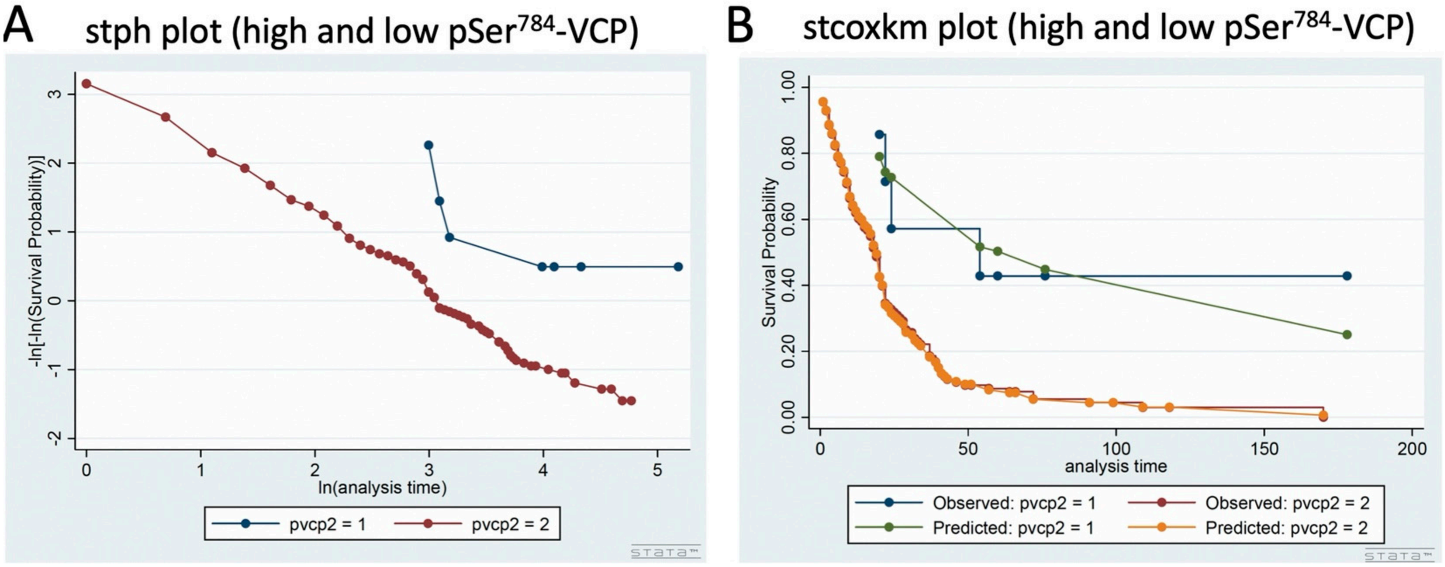


**Figure S2.** Visual assessment of proportional hazard assumption for pSer784-VCP, related to Figure 5. **(A)** The proportionality assumption was assessed visually using the –ln((−ln(survival)) plot. The subgroup curves of pSer784-VCP do not across, suggesting that the proportional hazards assumption is met. **(B)** The proportional hazards assumption was assessed for pSer784-VCP by comparing visually observed versus predicted survival curves. The observed and predicted survival curves accompany each other, suggesting that proportional hazards assumption is not violated.

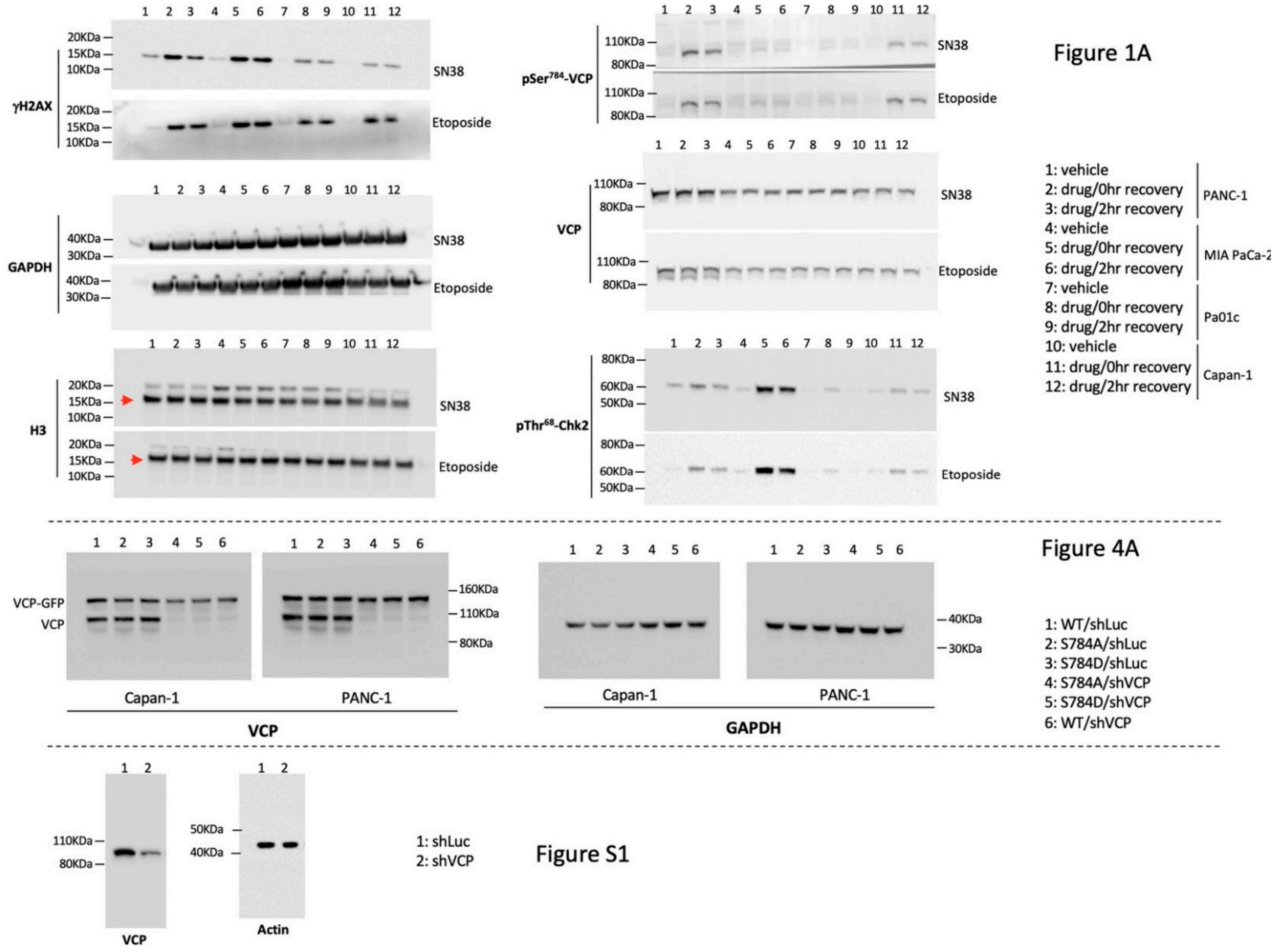


**Figure S3.** Raw Western blot data.

**Table S1.** Proportional hazard assumption analysis for pSer$^{784}$-VCP, related to Figure 5. 1. In this cox-regression model, the proportional hazards assumption was performed with the estat phtest command in Stata 12.0 and was not violated in any model (P>0.05, test carried out with the detail option in Stata 12.0 to examine the proportional hazards assumption for each predictor as well as to carry out the global test).

```
estat phtest, detail

    Test of proportional-hazards assumption

    Time:  Time
```

| | rho | chi2 | df | Prob>chi2 |
|---|---|---|---|---|
| pvcp2 | 0.06987 | 0.56 | 1 | 0.4531 |
| vcp | 0.03188 | 0.11 | 1 | 0.7441 |
| gender | -0.00842 | 0.01 | 1 | 0.9248 |
| race | 0.06178 | 0.45 | 1 | 0.5047 |
| tobacco | -0.01424 | 0.02 | 1 | 0.8863 |
| lymphatici~n | 0.01893 | 0.05 | 1 | 0.8272 |
| stage | 0.04645 | 0.34 | 1 | 0.5599 |
| venousinva~n | -0.02061 | 0.06 | 1 | 0.8125 |
| perineural~n | 0.02011 | 0.05 | 1 | 0.8278 |
| global test | | 2.07 | 9 | 0.9903 |
